# Glocalizing medical education through community clinical experiences: a model for socially accountable preclinical, community-based training

**DOI:** 10.3389/fmed.2026.1951514

**Published:** 2026-09-16

**Authors:** Rashida Daisley, Leslie S. Craig, Cordelle Lazare, Carlene Radix, Rhonda McIntyre

**Affiliations:** Community Medicine & Global Health, Office of External Affairs, Ross University School of Medicine, Two Mile Hill, St. Michael, Barbados

**Keywords:** community-academic partnerships, community-based medical education, glocalization, medical education, preclinical medical education, social accountability

## Abstract

Calls for “glocalization” in health professions education reflect growing expectations that global educational standards are meaningfully aligned with local priorities, in keeping with social accountability principles and shared values of relevance, equity, quality and cost-effectiveness. At the same time, increasing attention to the role of social determinants in shaping health outcomes challenges medical institutions to prepare practice-ready physicians who are culturally responsive and well-equipped to deliver holistic, patient-centered care. Scalable models that translate these priorities into structured, practice-oriented learning experiences remain limited within medical education, which is often constrained by traditional curricular structures, limited faculty capacity, and insufficient alignment between educational objectives and community realities. We describe a curricular service-learning model at Ross University School of Medicine that uses structured Community Clinical Experiences (CCEs) to connect globally relevant competency-based medical education with the health and social needs of the local community. We present core components of this model, including early, spiraled, clinical immersion, structured student reflection, and diverse social exposure, highlight their grounding in authentic community-academic partnerships, and outline key implementation strategies that support its adaptation and transfer across varied contexts. In so doing, we offer a potentially adaptable, scalable and sustainable approach for glocalized educational reform that is grounded in shared values while remaining relevant to different populations and health systems.

## Introduction

Increasing calls for “glocalization” in health professions education (HPE) signal growing expectations that academic institutions demonstrate social accountability by bridging global standards with local relevance (1–4). This outlook remains especially relevant within the context of globalized medical education, accreditation standards, and transnational medical schools, where educational systems must respond to local priorities while adhering to globally informed curricula (3, 5). However, despite widespread acceptance and endorsement of social accountability as a guiding principle, its operationalization in the design and implementation of educational models is less well described (6, 7). Clearer guidance is needed on how to design educational models that are adaptable, sustainable, and transferable across diverse settings while remaining aligned with the values of the World Health Organization (WHO) framework for medical school social accountability: relevance, equity, quality and cost effectiveness (8).

At the same time, widespread recognition of the role of social determinants of health (SDoH) in shaping health outcomes, challenges medical institutions to prepare practice-ready physicians who are culturally responsive and well-equipped to consider SDoH in the delivery of patient-centered care (9–11). Yet, prioritization of biomedical content in the preclinical years, coupled with a lack of congruence across the formal, informal and hidden curricula, often leaves these competencies insufficiently embedded within early medical training (12–15). Scalable models that translate these priorities into structured, practice-oriented learning experiences remain limited.

This perspective describes a curricular service-learning model at Ross University School of Medicine (RUSM) in Barbados, delivered through structured Community Clinical Experiences (CCEs) that contribute to health and social needs while intentionally integrating early, spiraled clinical exposure, diverse community engagement, and structured reflection into the medical education curriculum. This approach enhances professional identity formation and the development of key humanistic and clinical competencies. We present RUSM as a model for operationalizing glocalization by connecting globally relevant competency-based medical education (CBME) with the health and social needs of the local community.

### Community clinical experiences model: early, clinical immersion and spiraled, longitudinal exposure

CCEs are experiential clinical learning opportunities for basic science medical students and form a core component of the RUSM's medical school curriculum in the first 2 years. These experiences are designed to move the students beyond the traditional classroom setting to the community, where they receive early and consistent exposure to socially complex patients, underserved communities and the realities of healthcare systems. Unlike traditional clinical education environments, CCE sites are not hospital-based but include private and public primary care facilities, environmental health departments, specialist and urgent care clinics. Further, strategic community-academic partnerships (CAPs) extend learning and service opportunities beyond traditional clinical settings, bringing healthcare to marginalized communities and addressing local health priorities needs via targeted outreach in collaboration with community-based organizations (CBOs).

These CCEs are longitudinally integrated throughout the basic science curriculum, with clinical competencies progressively increasing in complexity as students advance through the semesters. During these sessions, students in groups of two (2) to six (6), are assigned to community-based preceptors who are appointed as adjunct clinical faculty and have been oriented to the program's goals and each semester's specific learning objectives. Prior to the commencement of each session, preceptors orient the students to the environs of the clinical site, its positioning in the wider Barbadian healthcare system and the patient populations that typically seek care at the facility. In the context of glocalized medical education, this is a critical step. Given the student population is largely comprised of students from the United States of America (US), the Barbadian healthcare system is conceptually foreign; therefore, meaningful engagement during CCEs require preceptor facilitated contextual and cultural awareness.

Following site orientation, students observe patient encounters, interview patients, and conduct focused clinical examinations under the guidance of the preceptor. Importantly, these encounters involve real, authentic patients seeking clinical care within community settings where students are placed, and not standardized or professional patients. Patient scenarios are then presented, and students engage in preceptor guided discussions centered around the clinical and sociocultural aspects of the case. Despite the growing use of simulation based clinical teaching, the value of real patient encounters should not be underestimated as it allows students to observe authentic patient care in the community setting, progressively strengthening their skills in communication, cultural competence and clinical reasoning (16). These CCEs are thus instrumental in molding the perspectives of future clinicians through a clear understanding of how culture, SDoH and health systems shape patient outcomes.

### Key pillars and implementation considerations for the CCE curricular model

As a model of glocalized CBME, CCEs offer a structured, scalable framework for integrating community needs into global medical education standards. This framework includes three (3) key pillars: early clinical immersion, diverse social exposures, and structured student reflection (Figure 1). Together, these pillars form a transferable model that can be adapted across contexts. While the specific community settings, partnerships, patient populations and priority health issues may differ according to local needs, the three (3) pillars represent core model elements and are considered essential for adoption. CAPs remain foundational, ensuring that the model is grounded in social accountability principles, and that CCE activities are responsive to community-identified needs and priorities. To illustrate how this model can be adapted and scaled, we outline its key pillars together with key implementation strategies and adaptation pathways that may be useful across varied contexts.

**Figure 1:**
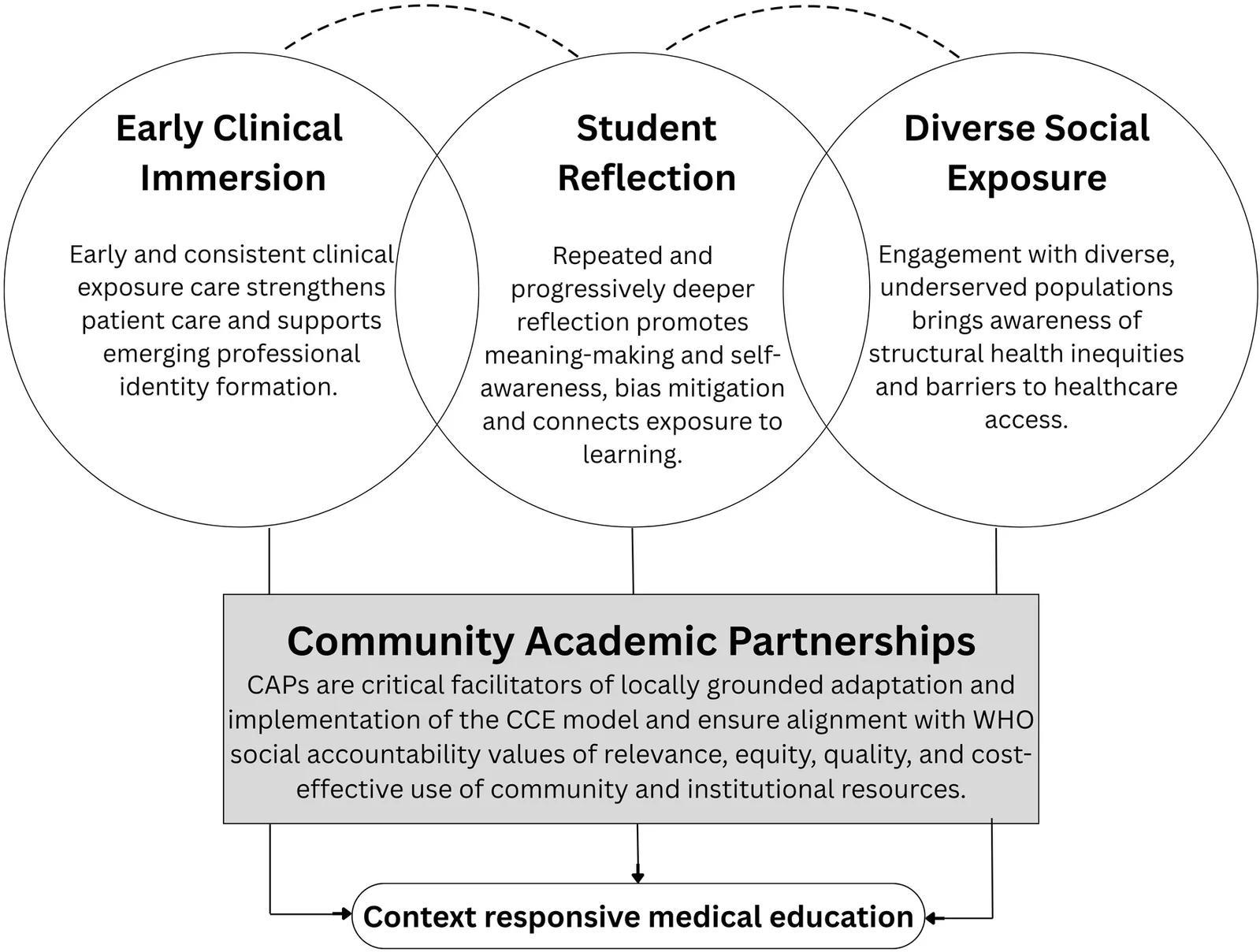
Structural framework of community clinical experiences (CCE) model.

### Early clinical immersion

Traditional medical education delineates curricula into pre-clinical components, where students develop competencies in basic science content, followed by clinical science training, that is characterized by application of this basic science knowledge to patient care (17, 18). This model presents an isolated view of biomedical knowledge which is disconnected from clinical care (19). The early community clinical immersion provided in CCEs contextualizes this theoretical, biomedical content, helping students to integrate this knowledge and apply it to clinical practice (18, 19). To facilitate this integration, curriculum developers should make the connection explicit for novice learners through relevant pre-reading assignments and clear signposting in lectures and/or community-based case discussions.

To effectively adapt and implement this model across HPE settings, an overarching goal of early clinical immersion needs to be clearly defined by curriculum leaders. In some circumstances, clinical reasoning competence is priority and structured bedside programs in hospital settings are most appropriate; however, the goal of the CCEs is broader than isolated clinical competence but also seeks to develop students’ sociocultural humility through a glocal lens (20). As such, this community preceptorship model is suitable to provide students with an authentic experience of the delivery of healthcare in underserved settings thereby making the impact of social factors such as food insecurity, structural inequality, health disparities and economic stability more tangible for novice learners. Through these CCEs, students’ perspective of quality of care is carefully shaped through the lens of social accountability, to reflect an understanding that quality care is not merely medically accurate but also culturally and context informed. While early clinical immersion reflects a key, and potentially transferable, model component that may be integrated into existing curricula without major redesign, the process is not without challenges and context-specific adaptation may be needed. Successful implementation requires careful alignment with learning objectives, institutional support and resources, and placement availability. Further, a spiraled approach is key to ensure that clinical exposures complement learning, and do not contribute to overload.

### Diverse social exposure

Diverse social exposure, the second pillar of the CCE model, allows students to directly engage with patients from a variety of backgrounds and strengthens their awareness of patients as persons living within complex social, structural, cultural and health systems contexts. Clinical and community sites are intentionally selected to expose students to the unique needs of specific underserved populations and are subdivided into the following categories: public primary care, private primary care and specialty clinics, environmental health sites and CBOs. Financed through taxation, Barbados provides universal healthcare for all citizens through a network of nine primary health centers positioned across key catchment areas in the island, called polyclinics, and one tertiary healthcare facility. The publicly funded health system is partially supported by private facilities where individuals pay out-of-pocket or through insurance. In the Caribbean context, persons of higher socio-economic status (SES) are more likely to utilize private healthcare compared to lower SES individuals; therefore, providing students with an opportunity to observe both settings ensures that they have a breadth of exposure to how individuals may enter healthcare systems (21).

The cornerstone of the social diversity pillar is the community outreach clinics delivered in partnership with key CBOs and in response to the needs of local community. Through community partners, outreach clinics are hosted in a variety of settings including community centers in rural and urban districts, places of worship and CBO facilities. CAPs and sustained relationships are thus key mechanisms for advancing social accountability goals and facilitating students’ exposure to diverse clinical and social contexts (22). Consistent with the WHO social accountability equity value, sustained partnerships with CBOs facilitate expansion of clinical placements and community-engaged opportunities, via pop-up clinics and a fully equipped clinical mobile unit, that bring services directly to underserved populations and address locally identified priorities. Namely, these partnerships include CBOs that provide chronic disease advocacy and support, sexual and reproductive health services for marginalized populations, social support for persons affected by homelessness, medical and developmental services for children with cognitive or learning disabilities, and healthcare and psychosocial support for gender and sexual minorities and persons living with sickle cell disease and autoimmune conditions.

Among the CCE model components thus far described, this pillar remains particularly amenable to context-specific adaptation, given varied clinical settings, local community priorities, patient populations, and available resources. Although the resource effectiveness of the CCE model has not been formally evaluated, its translation across settings should be guided by WHO's cost effectiveness value through alignment with available resources. Logistical considerations related to this component include placement coordination, student supervision, and establishing clear expectations regarding learner educational objectives and preceptor roles. Initial planning meetings, supported by ongoing communication, may help ensure consistency across partner sites. Furthermore, depending on the context, additional strategies, such as formal memoranda of understanding (MOUs), adjunct faculty appointments, remuneration mechanisms, or other forms of recognition, may also support partner engagement, coordination, and the sustained delivery of high-quality learning experiences.

### Structured student reflection

Although exposure to diverse communities can foster an awareness of unique social needs of certain patient populations, meaningful learning and attainment of global professional competency standards cannot be guaranteed with clinical encounters alone (23). Structured student reflection transforms passive exposure to intentional, purposeful learning through meaning-making and professional identity formation and are a critical component of the CCE model. After each community visit, students complete a reflective essay based on the encounter, guided by the *What? So what? Now what?* framework and a series of prompts that are aimed at inciting self-reflection and meaning-making (24). Reflective pieces are required components of each of the ten basic science modules, but their assessment contribution varies. In some modules, these essays are assessed using a *What? So what? Now what?* framework aligned rubric and contribute to the module's final assessment while, in others, minimum competency is a professional requirement aimed at emphasizing accountability. In formative stages of the curriculum, the goal of reflective practice is to shape students’ understanding of the SDoH, reflect on their own sociocultural biases and incorporate these perspectives into their own professional identity. Focus shifts in advanced preclinical semesters, where reflective practice is integrated into medical documentation encouraging students to make explicit connections between clinical care delivery and patients’ social context. This spiraled curricular approach leads to the progressive advancement of students’ cultural and structural competence.

In the context of glocalized HPE, structured reflection is a core feature of the model that should be retained. The reflective activity may be adapted to align with the local educational and clinical contexts while student assessments (e.g., reflective pieces and/or essays) may be standardized using common reflection prompts, rubrics and grading criteria to ensure comparable evaluation regardless of the clinical setting. Reflective practice facilitates student internalization of the social accountability principles by challenging students to critically consider how clinical care delivery reflects community health priorities, systemic access inequities, healthcare quality and the importance of cost-effective utilization of resources. There is a growing body of evidence that suggests health outcomes are largely shaped by these socioeconomic and cultural factors; thus, integration of this lens into clinical reasoning is especially important in medical education (25). Repeated and increasingly challenging review of these concepts as illustrated in Figure 2, facilitates adoption of a socially accountable lens into students’ professional identity. Reflection on these experiences is not merely descriptive but purposefully action oriented. After critical analysis of personal biases, cultural differences, social and structural determinants, students are challenged to conceptualize strategies to mitigate the impact of these factors on patient care in their future practice. In this way, structured reflection facilitates development of a socially accountable lens by moving students beyond mere descriptive analysis to how they might respond as future physicians. This triple loop learning model prompts students to challenge their existing mental models of patient centered care and use experiences such as those facilitated by CCEs to inform future actions in socially complex clinical encounters.

**Figure 2:**
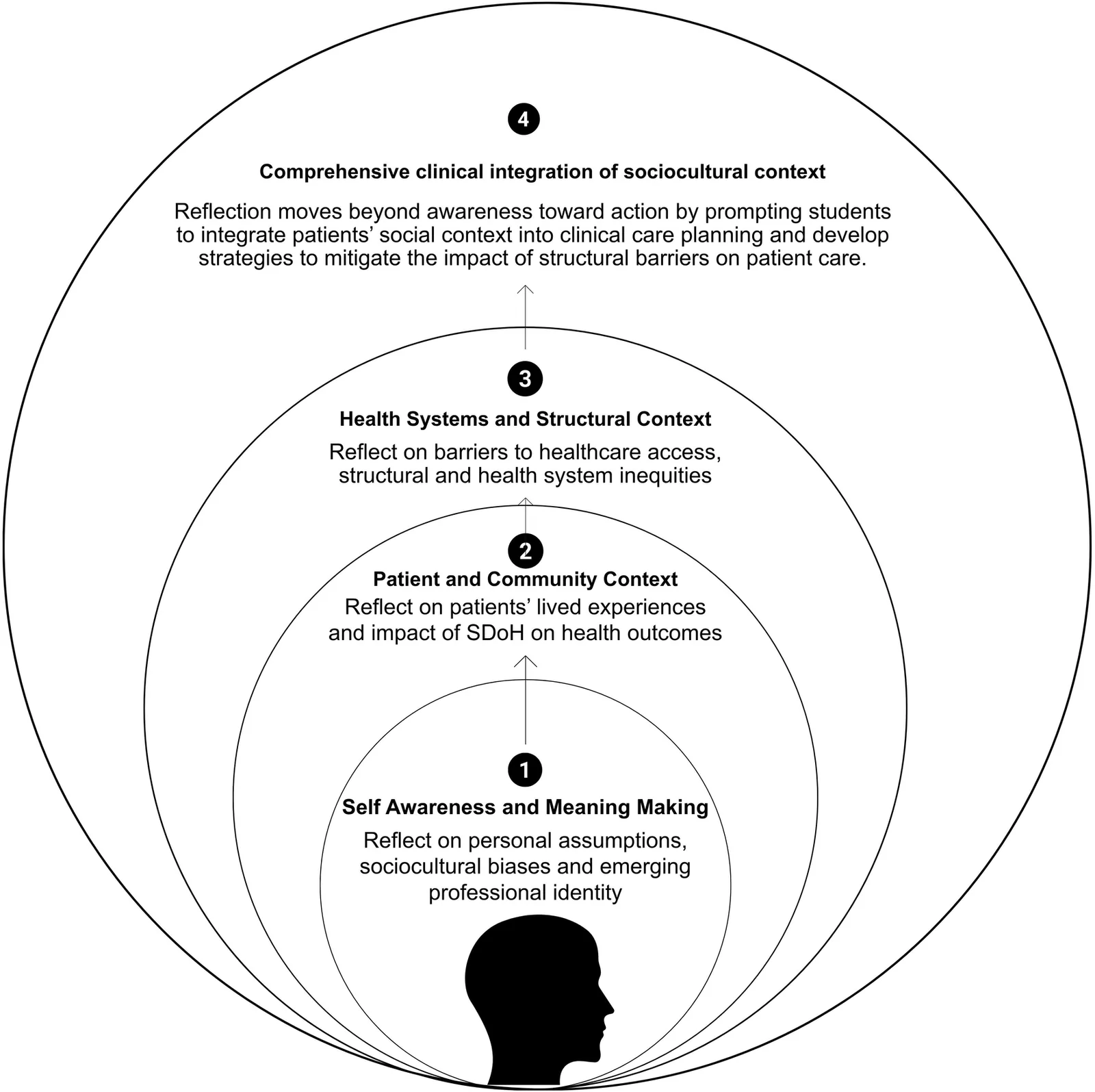
Structured reflection as a pathway to social accountability and professional identity formation.

### Foundation of glocalized medical education: community academic partnerships

Medical schools have an ethical responsibility to meaningfully contribute to the communities in which they exist through the lens of core values of relevance, equity, quality and resource efficiency (8). CAPs serve as the foundation for effective implementation of the CCE model and facilitate integration of social accountability across all three pillars by supporting each of these values.

Implementation of the CCE model into other HPE settings requires contextual adaptation, preservation of the core pillars and institutional support. Academic institutions should mirror the social accountability their students emulate by placing CAPs at the foundation of CCE adaptation. Authentic glocalization in clinical education needs to be developed in partnership with the community through meaningful engagement with NGOs, clinics and patients from populations of interest. Communities should be intentionally engaged to ensure that educational initiatives are relevant and designed to meet the local health priorities rather than simply being shaped by curricular needs. Cultivation of sustainable CAPs begins with assessing partnership readiness and compatibility. The findings of Andrews et al. (26) describe three (3) key indicators of readiness for collaborative research between CBOs and academic institutions which are applicable to clinical collaboration; these include goodness of fit, capacity and operations. To assess goodness of fit, both partners should examine to what extent their institutions have shared values, organizational culture, commitment and mutually beneficial intentions (26). Despite the value of goodness of fit, in the context of CCE adaption which are logistically complex at scale, assessing capacity and operations are critical. Potential CCE partners will be required to mobilize community members consistently and therefore should be well established as a trusted community leader which ensures they are able to access and communicate effectively with populations who often have distrust of academic institutions (26)

From an operational standpoint, potential partners should possess the personnel, space, time and resources to facilitate sustained partnership, given that sporadic or poorly organized initiatives violate the social accountability value of quality, potentially leading to discontinuity of care which can negatively impact the community and tarnish the reputation of supporting academic institutions (26). Quality should be intentionally incorporated by utilizing site preparation checklists and clinical protocols, and limiting the scope of clinical care to that which can be safely delivered within the community setting.

Following assessment of readiness, fostering trust between partners is paramount. Glocalized adaptation of this model will likely result in academic institutions working with underserved groups, who historically have experienced stigma and poor treatment when accessing healthcare and in academia. Therefore, mistrust and skepticism of academic institutions should be anticipated and mitigation strategies embedded within implementation plans. Trust requires clear, bidirectional, continuous and transparent communication. In practice, this can be cultivated through providing channels for regular feedback loops which allow all partners to express their views in a non-judgmental space (27). Power imbalances and tokenism are well known barriers to effective CAPs and perpetuate the abovementioned mistrust (28). To address this, partnerships should be mutually respectful and reciprocal where community members’ contributions and expertise are equally valued. In our setting, demonstration of appreciation through renumeration and dissemination of positive student feedback, further strengthens trust in partnerships.

To ensure that the core principle of glocalization, *act locally*, is ethically integrated, implementation of community-based initiatives should be preceded by a needs assessment to identify unique health priorities of the beneficiary populations. This ensures that educational initiatives such as pop-up clinics are designed to fill an existing gap in access and not simply tokenistic. Needs assessments should not be solely dependent on available statistics or authoritative figures but done in a collaboration with community representatives to identify undocumented community health needs. Qualitative data through stakeholder meetings provide rich insights into the unique, lived experience of people within the community and how culture shapes their perspective of health. Without these perspectives, health problems may be correctly identified but initiatives developed in response may be culturally inappropriate or disconnected from true local priorities (29). This approach is in keeping with the WHO values of relevance and equity, by ensuring alignment of CCE educational objectives and activities with the priority health needs of the beneficiary populations while also identifying populations experiencing barriers to clinical care and ensuring that service activities intentionally reach them. Alignment with quality and resource efficiency principles is further achieved by harnessing existing community resources and authentic partnerships that bring community knowledge and perspectives into the design and delivery of CCE activities, rather than relying exclusively on traditional healthcare system settings.

## Conclusion

Via early, spiraled, clinical exposure, structured student reflections and a diverse community-engaged framework that intentionally and authentically embeds CCEs within the preclinical curriculum, the RUSM model presented here aligns core medical education training with the social realities of health systems, community priorities and patient-centered care. This framework thus provides an approach to glocalized educational reform that balances shared goals (e.g., social accountability, community engagement, culturally competent physicians) with academic and accreditation-driven priorities, while enabling contextual adaptation and relevance to diverse contexts, populations and health systems. While this article describes the design, theory and conceptual rationale for the CCE model, future research using mixed methods approaches are needed to examine its impact to student learning outcomes, community benefit and to explore perspectives of community partners.

## Data Availability

The original contributions presented in the study are included in the article/Supplementary Material, further inquiries can be directed to the corresponding author.
